# Supplementary material for: Are boarding secondary schools suitable for students with asthma? An asynchronous online focus group discussion among members of an asthma awareness group
Source: PLoS One. 2024 Sep 6;19(9):e0304123. doi: 10.1371/journal.pone.0304123 (PMC11379369; doi:10.1371/journal.pone.0304123)
Supplement: Attachment — Submitted filename: PONE-D-23-13651_reviewerJBB.pdf [file pone.0304123.s001.pdf]

PLOS ONE

Are boarding secondary schools suitable for students with asthma? A focus group discussion

--Manuscript Draft--

Manuscript Number:	PONE-D-23-13651
Article Type:	Research Article
Full Title:	Are boarding secondary schools suitable for students with asthma? A focus group discussion
Short Title:	Are boarding secondary schools suitable for students with asthma?
Corresponding Author:	Kosisochi Chinwendu Amorha, Ph.D., FPCPharm<br>University of Nigeria<br>Nsukka, Enugu State NIGERIA
Keywords:	asthma; boarding school; Children; facilities; focus group; social media; WhatsApp
Abstract:	Introduction: Children in boarding schools spend most of their time without their parents, causing concerns about the suitability of such schools for children with asthma. This study assessed individuals' opinions regarding the suitability of boarding secondary schools for children with asthma.<br>Methods: A qualitative design was adopted for this study using a focus group discussion which held on a social media platform (WhatsApp®) of the Asthma Awareness and Care Group (AACG), a group only open to members. The discourse, involving 150 participants, was guided by a structured protocol. It was based on a case study comprising three questions. Data were analyzed via thematic analysis using framework principles.<br>Results: Out ofOf the 150 participants, there were 19 respondents (11 females and eight males). Majority of the respondents were aged ≤ 30 years (n = 17, 89.5%). The three main themes generated from the thematic analysis were: appropriateness of boarding school for children with asthma; facilities necessary for boarding schools to cater for same; and outright rejection of children with asthma by boarding schools. Respondents conceptualized the appropriateness of boarding schools for students with asthma in six distinct sub-themes: asthma severity and extent of control, child's self-efficacy and assertiveness, child equipped with tools (knowledge, inhalers, and asthma control diary), school awareness, facilities and active support, availability of a guardian, and the knowledge and perception of teachers and schoolmates about asthma. The sub- themes associated with the themes were presented, alongside exemplar quotes from respondents. Majority of the respondents were in support of allowing children with asthma go to a boarding school but with some caveats such as without liability to the school, if facilities are unavailable.<br>Conclusion: Children's age, autonomy, asthma management status, and the school's readiness were identified as important considerations for the safe attendance of children with asthma at boarding schools.
Order of Authors:	Kosisochi Chinwendu Amorha, Ph.D., FPCPharm
	Kossy Maryann Ochie, B.Pharm
	Stephen Chukwuma Ogbodo, B,Pharm, M.Sc.
	Olisaemeka Zikora Akunne, B.Pharm
	Ogechi Christiana Obi, B.Pharm
	Nwamaka Theresa Ene, B.Pharm
	Chukwudi Richard Ifeanyi, B.Pharm
	Jonathan Ikokwu, B.Pharm
	Chibuike Victor Eze, B.Pharm
	Emmanuella Tochukwu Ogbonna, B.Pharm
	Marydith Ifeoma Chukwu, B.Pharm

Commented [u1]: The n=17 does not add up to the 150. Please, clarify your statement and make it easy to understand, reveling where the remaining n=133 belongs.

Powered by Editorial Manager® and ProduXion Manager® from Aries Systems Corporation

	Chinedu Collins Okafor, B.Pharm
	Chiamaka Ruth Echeta, B.Pharm
	Somtochi Prosper Nwani, B.Pharm
	Christabel Ogechukwu Okoye
	Vanessa Chinweike Okonkwo
	Chisom Jennifer Eneje, B.Pharm
	Gerald Obinna Ozota, B.Pharm
Additional Information:	
Question	Response
Financial Disclosure<br><br>Enter a financial disclosure statement that describes the sources of funding for the work included in this submission. Review the submission guidelines for detailed requirements. View published research articles from PLOS ONE for specific examples.<br><br>This statement is required for submission and will appear in the published article if the submission is accepted. Please make sure it is accurate.<br><br>Unfunded studies<br>Enter: The author(s) received no specific funding for this work.<br><br>Funded studies<br>Enter a statement with the following details:<br>• Initials of the authors who received each award<br>• Grant numbers awarded to each author<br>• The full name of each funder<br>• URL of each funder website<br>• Did the sponsors or funders play any role in the study design, data collection and analysis, decision to publish, or preparation of the manuscript?<br>• NO - Include this sentence at the end of your statement: The funders had no role in study design, data collection and analysis, decision to publish, or preparation of the manuscript.<br>• YES - Specify the role(s) played.<br><br>* typeset	The authors received no specific funding for this work
Competing Interests	The authors have declared that no competing interests exist.

Use the instructions below to enter a competing interest statement for this submission. On behalf of all authors, disclose any competing interests that could be perceived to bias this work—acknowledging all financial support and any other relevant financial or non-financial competing interests.<br><br>This statement is required for submission and will appear in the published article if the submission is accepted. Please make sure it is accurate and that any funding sources listed in your Funding Information later in the submission form are also declared in your Financial Disclosure statement.<br><br>View published research articles from PLOS ONE for specific examples.<br><br>NO authors have competing interests<br><br>Enter: The authors have declared that no competing interests exist.<br><br>Authors with competing interests<br><br>Enter competing interest details beginning with this statement:<br><br>I have read the journal's policy and the authors of this manuscript have the following competing interests: [insert competing interests here]<br><br>* typeset	
Ethics Statement<br><br>Enter an ethics statement for this submission. This statement is required if the study involved:<br><br>• Human participants<br>• Human specimens or tissue<br>• Vertebrate animals or cephalopods<br>• Vertebrate embryos or tissues<br>• Field research	N/A

Powered by Editorial Manager® and ProduXion Manager® from Aries Systems Corporation

Write "N/A" if the submission does not require an ethics statement.

General guidance is provided below. Consult the submission guidelines for detailed instructions. Make sure that all information entered here is included in the Methods section of the manuscript.

Format for specific study types

Human Subject Research (involving human participants and/or tissue)
- Give the name of the institutional review board or ethics committee that approved the study
- Include the approval number and/or a statement indicating approval of this research
- Indicate the form of consent obtained (written/oral) or the reason that consent was not obtained (e.g. the data were analyzed anonymously)

Animal Research (involving vertebrate animals, embryos or tissues)
- Provide the name of the Institutional Animal Care and Use Committee (IACUC) or other relevant ethics board that reviewed the study protocol, and indicate whether they approved this research or granted a formal waiver of ethical approval
- Include an approval number if one was obtained
- If the study involved non-human primates, add additional details about animal welfare and steps taken to ameliorate suffering
- If anesthesia, euthanasia, or any kind of animal sacrifice is part of the study, include briefly which substances and/or methods were applied

Field Research

Include the following details if this study involves the collection of plant, animal, or other materials from a natural setting:
- Field permit number
- Name of the institution or relevant body that granted permission

Powered by Editorial Manager® and ProduXion Manager® from Aries Systems Corporation

Data Availability<br><br>Authors are required to make all data underlying the findings described fully available, without restriction, and from the time of publication. PLOS allows rare exceptions to address legal and ethical concerns. See the PLOS Data Policy and FAQ for detailed information.<br><br>A Data Availability Statement describing where the data can be found is required at submission. Your answers to this question constitute the Data Availability Statement and will be published in the article, if accepted.<br><br>Important: Stating 'data available on request from the author' is not sufficient. If your data are only available upon request, select 'No' for the first question and explain your exceptional situation in the text box.<br><br>Do the authors confirm that all data underlying the findings described in their manuscript are fully available without restriction?	Yes - all data are fully available without restriction
Describe where the data may be found in full sentences. If you are copying our sample text, replace any instances of XXX with the appropriate details.<br><br>• If the data are held or will be held in a public repository, include URLs, accession numbers or DOIs. If this information will only be available after acceptance, indicate this by ticking the box below. For example: All XXX files are available from the XXX database (accession number(s) XXX, XXX.).<br>• If the data are all contained within the manuscript and/or Supporting Information files, enter the following: All relevant data are within the manuscript and its Supporting Information files.<br>• If neither of these applies but you are able to provide details of access elsewhere, with or without limitations, please do so. For example:	All relevant data are within the manuscript.

Powered by Editorial Manager® and ProduXion Manager® from Aries Systems Corporation

Data cannot be shared publicly because of [XXX]. Data are available from the XXX Institutional Data Access / Ethics Committee (contact via XXX) for researchers who meet the criteria for access to confidential data.<br><br>The data underlying the results presented in the study are available from (include the name of the third party and contact information or URL).<br>• This text is appropriate if the data are owned by a third party and authors do not have permission to share the data.<br><br>* typeset	
Additional data availability information:	

Powered by Editorial Manager® and ProduXion Manager® from Aries Systems Corporation

Are boarding secondary schools suitable for students with asthma? A focus group discussion

Kosisochi Chinwendu Amorha [1, 2, 3], Kossy Maryann Ochie [3, 4], Stephen Chukwuma Ogbodo [3, 5], Olisaemeka Zikora Akunne [3, 6], Ogechi Christiana Obi [3, 7], Nwamaka Theresa Ene [3, 8], Chukwudi Richard Ifeanyi [3, 9], Jonathan Ikokwu [3, 10], Chibuike Victor Eze [1, 3], Emmanuella Tochukwu Ogbonna [1, 3], Marydith Ifeoma Chukwu [3, 11], Chinedu Collins Okafor [3, 12], Chiamaka Ruth Echeta [1, 3], Somtochi Prosper Nwani [3, 13], Christabel Ogechukwu Okoye [1, 3], Vanessa Chinweike Okonkwo [1, 3], Chisom Jennifer Eneje [1, 3], Gerald Obinna Ozota [1, 3]

1. Department of Clinical Pharmacy and Pharmacy Management, Faculty of Pharmaceutical Sciences, University of Nigeria Nsukka, PMB 410001, Enugu State, Nigeria.

2. Department of Clinical Pharmacy and Pharmacy Practice, Faculty of Pharmaceutical Sciences, Bingham University, PMB 005, Karu, Nasarawa State, Nigeria.

3. Asthma Awareness and Care Group (AACG), https://www.asthmacaregroup.org.ng

4. Department of Clinical Pharmacy and Pharmacy Management, Nnamdi Azikiwe University, PMB 5025, Awka, Anambra State, Nigeria

5. Pharmacy Department, Enugu State University Teaching Hospital, Nigeria

6. ASK Medical and Diagnostic Center, Federal Capital Territory, Abuja, Nigeria

7. Federal Medical Centre, PMB 7001, Umuahia, Abia State, Nigeria

8. Kareda Pharmacy, Asaba, Delta State, Nigeria

9. National Institute for Pharmaceutical Research and Development, PMB 21, Abuja, Nigeria

10. Pharmacy Council of Nigeria, Lagos Zonal Office, PMB 415, Yaba, Lagos State, Nigeria

11. Federal Medical Center, PMB 1001, Lokoja, Kogi State, Nigeria

12. Lyn Edge Pharmaceuticals Limited, Lagos, Nigeria

13. Medplus Pharmacy Nigeria Limited, Lagos, Nigeria

*** Corresponding Author:**

Email: kosisochi.amorha@unn.edu.ng

Contributorship:

Conceptualization/Study design: Amorha, K. C.

Literature search/Manuscript preparation: All authors [*Abstract:* Ene, N.T. and Nwani, S.P; *Introduction:* Obi, O. C., Ogbonna, E. T., Okoye, C. O., and Eneje, C. J.; *Methods:* Ifeanyi, C. R., Chukwu. M. I., Ozota, G. O., Okonkwo, V. C.; *Discussion, Results and Conclusion:* Akunne, O. Z., Eze, C. V., Ikokwu, J., and Okafor, C. C.; *Transcribing of Results and Analysis:* Ochie, K. M., Ogbodo, S. C., and Echeta, C. R., Amorha, K. C.]

Analysis of data: Ochie, K. M., Ogbodo, S. C., and Echeta, C. R., Amorha, K. C.

Review of manuscript: Amorha, K. C., Ochie, K.M., Ogbodo, S. C.

All authors read and approved the final manuscript for publication.

Abstract

Introduction: Children in boarding schools spend most of their time without their parents, causing concerns about the suitability of such schools for children with asthma. This study assessed individuals' opinions regarding the suitability of boarding secondary schools for children with asthma.

Methods: A qualitative design was adopted for this study using a focus group discussion which held on a social media platform (WhatsApp®) of the Asthma Awareness and Care Group (AACG), a group only open to members. The discourse, involving 150 participants, was guided by a structured protocol. It was based on a case study comprising three questions. Data were analyzed via thematic analysis using framework principles.

Results: **Out of** ~~Of~~ the 150 participants, there were 19 respondents (11 females and eight males). Majority of the respondents were aged ≤ 30 years (n = 17, 89.5%). The three main themes generated from the thematic analysis ~~were~~ include: appropriateness of boarding school for children with asthma; facilities necessary for boarding schools to cater for same; and outright rejection of children with asthma by boarding schools. Respondents conceptualized the appropriateness of boarding schools for students with asthma in six distinct sub-themes: asthma severity and extent of control, child's self-efficacy and assertiveness, child equipped with tools (knowledge, inhalers, and asthma control diary), school awareness, facilities and active support, availability of a guardian, and the knowledge and perception of teachers and schoolmates about asthma. The sub-themes associated with the themes were presented, alongside exemplar quotes from respondents. Majority (%) of the respondents were in support of allowing children with asthma go to a boarding school but with some caveats such as without liability to the school, if facilities are unavailable.

Commented [u2]: The number of the respondents in this research are not sufficient in my own opinion for reasonable scientific conclusion.

Why were more people not recruited to give a wider population view?

If it were the other way round, 131 respondents and 19 non respondents, it will be more reasonable to make some conclusions on this important study

Commented [u3]: It is important to quantify this majority to give a clear statistical picture of the said majority

Conclusion: Children's age, autonomy, asthma management status, and the school's readiness were identified as important considerations for the safe attendance of children with asthma at boarding schools.

Key Words: asthma; boarding school; children; facilities; focus group; social media; WhatsApp

Commented [u4]: Aren't these two the same thing? Aren't the two social media?

Introduction

Childhood asthma, recognized as the most common chronic respiratory disease among children, poses the highest disability burden in this population and accounts for most cases of school absenteeism [1, 2]. Asthma is a multifactorial disease caused by a combination of genetic, host, and environmental factors [3,4]. The global prevalence of asthma has increased significantly in the last forty years, with the World Health Organization (WHO) estimating that 300 million people currently live with asthma, and this is projected to reach 400 million by 2025 [1]. In terms of mortality, 250,000 people die from asthma annually, with a reported mortality rate of up to 0.7 per 1000 among children [1,5].

Nigeria has one of the highest burdens of childhood asthma in Africa, with a rapidly increasing prevalence from 10.7% in 1999 to about 20% in 2014 [6]. Since basic education is a legal right in Nigeria, as with most other countries, the management of asthma among children within the school system has become a central issue [7]. The Global Initiative for Asthma (GINA) estimated that 14% of school-aged children have had at least one episode of wheezing in the past 12 months [8]. For children attending "day" schools, their parents have ample opportunity to monitor and manage their asthma, as they return home, daily, after school. This is less feasible for boarding school children who enjoy significantly reduced parental oversight. Hence, boarding school children with asthma often have to take up greater responsibility for managing their triggers, symptoms, and attacks alone or with the help of the school management. As such, many parents/guardians of children with asthma may be skeptical about sending their children to boarding schools.

It has been documented that children with asthma face significant challenges in school which can affect their academic performance and quality of life [7]. Acute asthma attacks in school children accounts for 2-10% of emergency hospital visits in Nigeria, largely due to the fact that most

schools lack appropriate facilities and health services to manage asthma episodes [9,10]. Students with asthma may also experience stigmatization through exclusion from school programs like sports and other physical activities [11, 12]. Although studies have shown that incorporating asthma-related topics in school curriculum will improve students' and teachers' knowledge of asthma and improve the experience of children with asthma, parents and caregivers are still faced with the dilemma of the suitability of boarding schools for their children with asthma [13,14].

Thus, this study was designed to evaluate individual perceptions and opinions regarding the appropriateness of boarding schools for students with asthma, particularly in Nigeria. It further aimed to elicit perspectives about the facilities, appropriate trainings, and conditions that would enable the effective management of students with asthma in boarding schools.

Methods

Study design~~/setting~~

This was a qualitative study involving a focus group discussion which held on the social media platform (WhatsApp®) of the Asthma Awareness and Care Group (AACG). The discourse, which held on 22 March, 2023, was guided by a structured protocol. It was based on a case study comprising three questions. The case study was posted in the Group, without previous publicity. Members of the Group were informed that only comments received within a specified time frame would be utilized for the study (14:00 – 21:00 hours). A 7-hour time frame was fixed, considering that the discussion was impromptu. The discourse was moderated by the Founder of the Group. AACG is a team of asthma enthusiasts who aim to improve asthma awareness and care, in communities [15].

Commented [u5]: What kind of method is this and who invented or modified it for use in research such as this?

Commented [u6R5]:

Ethical approval

The study did not require ethical approval. It was a focus group discussion conducted online, in a closed group on WhatsApp®. The group is restricted to members of the Asthma Awareness and Care Group (AACG). Participation of members of the Group was voluntary. Members who participated were informed that their comments would be extracted for research purposes. They were also informed that the authors would be members of AACG who could identify individual participants during or after data collection.

Eligibility criteria

Eligibility criteria comprised members of the Asthma Awareness and Care Group (AACG) who were registered on the AACG WhatsApp® platform. As of the time the study was conducted, there were 150 registered members on the AACG WhatsApp® platform. Members of the Group were majorly Pharmacists (n = 142, 94.7%), with a large proportion having not more than 10 years of experience. The Pharmacists practiced in community, hospital, academia, industry, and administrative pharmacy settings. Other members of the Group included: five undergraduate pharmacy students, one Public Relations Professional, one Economist, and one Mechanical Engineer. Only comments received within the 7-hour time frame were included.

Commented [u7]: What is the rationale behind this time restriction?

Sample size and selection

Sample size was not calculated. All registered members of the AACG WhatsApp® platform were eligible for participation. Convenience sampling technique, within a specified timeline, was employed. Comments were received from 19 participants.

Commented [u8]: Why was sample size not determined/calculated?

How well aware are people of the AACG in order for them to have joined, so that the chances of participants and respondents could increase?

Data collection

This case study was posted in the AACG WhatsApp® platform:

AB is an 11-year-old known patient with asthma.

As the closest healthcare provider to her family:

1. Would you advise AB to go to a Boarding School? Kindly justify your position.

2. What facilities would you advise Boarding Schools have, to cater for students with asthma?

3. Should Boarding Schools simply reject patients with asthma i.e., better safe than sorry?

The comments from the participants provided data for the study. Demographic data were collected for each respondent.

Data analysis

After the group discussion, a thematic analysis was conducted using framework analysis principles and generally adhering to the five stages of familiarization: identifying a thematic framework, indexing, charting, mapping, and interpretation. Data collected were analyzed using themes, sub-themes, and quotes from the participants' comments. In each instance, nine researchers independently acquainted themselves with a subset of the data and assigned codes to portions of text. The codes were used to generate the initial sub-themes, which were then grouped into higher-order themes through an iterative process. Discrepancies between the nine researchers' frameworks were resolved through discussion. At team meetings, the addition or development of themes, subthemes, and quotes during the indexing process were discussed. When charting the data by theme, subtheme, and quote, similarities and differences between the datasets were considered after the initial analysis. The predefined themes were investigated, and an inductive method was used to derive quotations and subthemes from the primary themes. During the process of coding, a 'tree structure' was generated in which themes, subthemes, and quotes were connected. The findings were presented, based on the themes and subthemes.

Results

Nineteen respondents participated in the focus group discussion, comprising 11 females and eight males. Majority of the respondents were aged ≤ 30 years (n = 17, 89.5%), Table 1.

Table 1: Socio-demographic characteristics of the participants, N = 19

Participant Number	Gender	Age category (years)
P1	F	26 – 30
P2	M	26 – 30
P3	M	26 – 30
P4	F	18 – 25
P5	M	26 – 30
P6	M	26 – 30
P7	F	18 – 25
P8	F	26 – 30
P9	M	26 – 30
P10	F	26 – 30
P11	F	18 – 25
P12	F	18 – 25
P13	F	26 – 30
P14	M	> 30
P15	F	26 – 30
P16	F	26 – 30
P17	M	26 – 30
P18	M	> 30
P19	F	18 – 25

M = Male; F = Female;

All respondents were Pharmacists, except P11 and P12 who were in undergraduate pharmacy school

The thematic analysis produced three themes: the appropriateness of boarding school for children with asthma (Table 2); the facilities necessary for boarding schools to cater for children with asthma (Table 3); and the outright rejection of children with asthma by boarding schools (Table 4).

Respondents conceptualized the appropriateness of boarding schools for students with asthma according to six distinct sub-themes: asthma severity and extent of control, child's self-efficacy and assertiveness, child equipped with tools (knowledge, inhalers, and asthma control diary), school awareness, facilities and active support, availability of a guardian, and the knowledge and perception of teachers and schoolmates about asthma.

The subthemes associated with each of these themes are presented, alongside exemplar quotes from respondents.

Table 2: Appropriateness of boarding schools for students with asthma

Theme	Subthemes	Quotes
Appropriateness of boarding schools for students with asthma	1. Asthma severity and extent of control	(a) “I would advise AB to go to a boarding school if her asthma is well-controlled. In general, I would judge the severity of her condition”.
		(b) “It would depend on her level of asthma control. That she is a known patient with asthma does not necessarily imply that she has poorly-controlled asthma. It is possible that she might have well-controlled or even perfectly-controlled asthma”.
		(c) “Making sure AB stays safe with asthma at a boarding school can feel a bit daunting. However, I would advise AB to go to a boarding school if her asthma is well-controlled”.
		(d) ‘Since AB is a known asthma patient, I want to assume that her parents have placed her on the right medications. If she is properly

		managed, that means if her Asthma Control test is 20 and above, she can go to the boarding school”.
	2. Child’s self-efficacy and assertiveness	(a) “Has AB been taught how to stand up for herself with regards to her health status, respectfully but firmly? For example, she can say "Sorry I can’t participate in sweeping because it is too dusty and I’m allergic to dust”.
		(b) “AB’s ability to take care of herself without supervision should be considered. AB’s sense of responsibility and knowledge about her health condition should be considered too. Checking all of these boxes are huge expectations from an 11-year-old, even in the best-case scenarios”.
	3. Child equipped with tools: knowledge, inhalers, asthma diary	(a) “She can go but her parents must equip her with her inhaler, asthma control diary and other things she needs to be successful while in school”.

		(b) "AB can be advised to go to a boarding school if there's proper knowledge about her asthma triggers, extent of exacerbation, and proper management plan".
Appropriateness of boarding schools for students with asthma		(c) "The child should become conversant with managing her condition and avoiding her triggers and not always needing external help because she won't remain a child".
		(d) "Irrespective of the level of her asthma control, it is important to find out if she knows her asthma triggers and her level of asthma self-efficacy. At 11, and with a history of asthma, her knowledge of asthma self-management will need to be brought to the fore in a boarding school".
	4. School awareness, facilities and active support	(a) 'I will also go to inform the school authorities of her condition and her dos and don'ts".
		(b) "Yes, I would advise AB to go to a boarding school because

the one I went to, made sure we submitted our blood test results

		and other health challenges before we were admitted. The school also had the Red Cross Society in charge of administering First Aid in case of any Emergency”.
		(c) “Boarding schools can be safe and appropriate environments for students with asthma if they have adequate facilities and support systems in place to manage their condition.”
Appropriateness of boarding schools for students with asthma	School awareness, facilities and active support (Continued)	(d) “Though I'd say it's okay to take the child to a boarding school if the condition is not so severe, as long as the school has what it takes to manage asthmatic situations when it arises.”
	5. Availability of a guardian	(a) “But in a case where she doesn't have a proper knowledge about the condition, the parents should hand her over to a guardian or tell the school authorities. I can vividly remember, then in school, asthmatic students were given some kind of preferential treatment, like bathing in hot water, not allowed to run or even do some manual labours and so on”.

		(b) “If being in a boarding school is necessary, then it is okay for her to go. However, her parents would have to assign a guardian to check with her regularly”.
	6. Knowledge and perception of teachers and schoolmates about asthma	(a) “We also knew those who were Asthmatic, and in case of an emergency, you knew who to ask for an inhaler during crises”
		(b) “Students should also be enlightened on what to do when someone has a crisis”.
	7. Boarding school not advised	(a) “If there’s any chance at all that AB can go to school from home, then AB shouldn’t consider a boarding school at such a young age”.
		(b) “AB is young. Triggers for asthma attacks are peculiar to individuals. It’s possible that most of AB’s triggers have not even been known, the change of environment, coupled with the emotional unrest that comes with moving into a boarding school could all be triggers on their own for such a young patient. It’s going

to be a whole new world for AB in a boarding school, with exposure

Appropriateness of boarding schools for students with asthma	to a lot of foreign factors/allergens. An 11 y/o wouldn't be able to properly manage all of it".
	(c) "I would NOT advise AB to go to a boarding school because AB is still young and may not know how to take care of herself without proper supervision. Also, AB's triggers might not be known, completely".
	(d) "I would not advise an 11-year-old patient with asthma to go to a boarding school because; I feel she's still very tender and may not be able to deal with the condition, alone, in a boarding school. The living conditions in most boarding schools may not be suitable for her health. Being that she may not have developed adequate understanding and awareness of his condition (at her age), she may not know how to identify her triggers and avoid them. Even if she does know the triggers, what if she can't avoid them, given that it's a public home?"

	Boarding school not advised (Continued)	(e) “I would not advise AB to go to a boarding school. That is a young age for a child with asthma to be in boarding school whether the asthma is well-controlled or not. Triggers can happen anytime and I believe at that age the child might not be fully aware of all the them. Even adults get triggered at any point, much less an 11-year-old trying to adjust to a new environment.”
		(f) “My cousin was in a boarding school but she recently had to leave because her asthma was not properly managed. As long as there are alternative day-schools that are good, I don’t see the reason why the child should be put at risk at such a young age”.
Appropriateness of boarding schools for students with asthma		(g) “Most asthma-related deaths occur in low- and lower-middle-income countries, where under-diagnosis and under-treatment is a challenge- (WHO) and I believe that the best way to prevent these complications is to avoid asthma triggers and this is often difficult in boarding schools where there are several shared facilities and

	spaces than in day school. There is a higher chance of disease spreading such as flu which could trigger attacks”.
	(h) “Part of the reason why parents send their kids to boarding schools is to help them live independently but an 11-year-old with asthma away from their parents might only lead to a lot of micromanaging which in this case defeats the aim. The child is better off in a day school where they can be monitored daily at home which by research is recommended”.

Table 3: Facilities necessary for boarding schools to cater for students with asthma

Theme	Subtheme	Quotes
	1. Adequate and functional on-site medical facilities with round the clock availability of health professionals e.g., nurses	(a) "They must have a functional sick bay with a trained nurse on standby and the sick bay must be stocked with inhalers and even nebulizers in cases of emergency".
		(b) "It will also be necessary to know if the school has health professionals in their sick-bay or clinic, 24/7, who have the knowledge and expertise (as well as medications and devices), to act as responders if there is severe asthma exacerbation".
Facilities necessary for boarding schools to carter for students with asthma		(c) "A sick bay with a trained nurse with empathy because ours was trained but lacked empathy".
		(d) "Boarding schools should have trained health personnel with updated knowledge of asthma management. They should have a clinic equipped with asthma relievers to help manage any asthma episodes and emergencies".

		(e) "A boarding school should have emergency asthma kits such as nebulizers, inhalers and oxygen devices. Also, they should have an ambulance for carrying patients when referred".
		(f) "They should have a medical staff with proper knowledge and orientation about asthma intervention and management, an equipped drug store with essential asthma medications and inhalers, and a sick bay".
Facilities necessary for boarding schools	2. Effective referral system for severe emergencies.	(a) "The school must be affiliated with a nearby hospital where they can rush the students to, in cases of emergency like status asthmaticus".
		(b) "There should be a retainership agreement with a standard hospital or a Pulmonologist to manage serious cases before the physical arrival of parents/guardians".
		(c) "The school should have a hospital subsidiary to facilitate referral systems in severe emergencies".

to carter for students with asthma	3. Measures to minimize exposure to environmental triggers: dust, pollen, cold.	(a) “The school should take note of the students with asthma and ensure as much as possible that they are not exposed to chores that could trigger an attack”.
		(b) “Boarding schools should have facilities such as clean and well-ventilated dormitories, classrooms, and common areas to reduce exposure to triggers such as dust, molds, and pet dander”.
		(c) “A boarding school can be safe and able to cater for asthmatic students if it has less exposure to most common allergens including dust and pets, clear routines that limit allergen induction like controlled fumigation practice especially during holidays when students are away”.
	4. Collaboration and communication between parents, school, guardian and the child	(a) “It is also important for schools to have clear communication with parents or guardians about their child's asthma care plan, medication requirements, and emergency contact information”.

		(b) “It’s very paramount that the school and the parents work as partners; ensuring proper communication, provision of medications and necessary resources to keep children with asthma, safe in school”.
Facilities necessary for boarding schools to carter for students with asthma	5. Training for staff and students to recognise asthma signs and help	(a) “They should have trained staff who are familiar with asthma management, including recognizing symptoms, administering medication, and responding to emergencies”.
		(b) “The school should have a Red Cross Society which is an organization of students like you who are continuously trained on various crisis situations that may arise and how to deal with it”.
		(c) “Have teachers and management who are not just aware but have positive perceptions about asthma (You wouldn't want to send a child to a school where the teachers or management believes that asthma is infectious or that asthmatic attacks are solved by persons surrounding the asthmatics and pouring him/her water)”.

(d) “Staff of the school should be First Aid-trained and aware of what to do in an asthma attack and how to use a reliever inhaler. Further training can be offered to individual staff that are supporting children to use their inhalers”.

Table 4: Outright rejection of children with asthma by boarding schools

Theme	Subthemes	Quotes
Outright rejection of children with asthma by boarding schools	1. Never reject – to avoid discrimination and stigmatization	(a) “No, boarding schools should not simply reject students with asthma. Asthma is a common chronic condition, and with proper management and support, students with asthma can thrive academically and socially. Rejecting students with asthma is discriminatory and can limit their opportunities for personal and academic growth. Instead, boarding schools should work to create an inclusive and supportive environment for all students, including those with asthma. This can include providing appropriate facilities and resources, as well as promoting education and awareness about asthma among students, staff, and faculty”
		(b) “No! It could be misconstrued as stigmatization”.
	2. Reject if unprepared - better safe than sorry	(a) “Only if they are not equipped. I think it's best for everyone if ill-equipped boarding schools reject known asthmatics. I can

	understand why other colleagues feel differently, as "rejection" has strong connotations of discrimination and unequal opportunity. But, *better safe than sorry"*!
	(b) "Refusing a patient because they have asthma can be termed discriminatory. However, if they are not equipped, accepting such a child is simply criminal".
	(c) "A boarding school should not reject a student with asthma. They can do so if they know fully well, they are not properly equipped and can't handle the situation".
3. Accept even if unprepared but without liability to the school	(a) "A boarding should not outrightly reject a student as this can be a form of stigma, they should focus on been equipped but if all these are not available, it is best they are clear on the extent of service they can offer for such emergency to the parent or guardian and the decision left on them to make if they would want to put their child or ward or not".

	Accept even if unprepared but without liability to the school (Continued)	(b) “They should advise parents to opt for day school. However, if parents insist school should not be held liable”.
Outright rejection of children with asthma by boarding schools	4. Accept if prepared	(a) “Where boarding schools should not outrightly reject an asthma student, they must ensure that they are fully equipped to take care of those children”.
		(b) “Boarding schools should not reject patients as long as they have a good facility to take care of them to avoid asthma being like a social stigma”.
		(c) “No, boarding schools should not reject all asthma patients. What they can do is to make sure that the patient clearly informs the school and staff that needs to know and that the patient is properly managed. Make sure that the patient is compliant with medications and safety practices.”

Discussion

The findings of this study address the issue of appropriateness of boarding schools for children with asthma. The respondents were stakeholders involved in asthma care and knowledgeable about the resources necessary for adequate management of asthma.

Theme 1: Appropriateness of boarding schools for children with asthma

The question of whether or not boarding schools are appropriate for students with asthma is a complex issue that requires careful consideration of several factors. The opinions expressed by the respondents indicate that there is no clear consensus on the matter and that the decision should be based on a case-by-case evaluation.

The majority of participants opined that children with asthma can be allowed to attend boarding schools if there are appropriate and favourable conditions which include: that their asthma is well-controlled, they are well-equipped by their parents on skills for asthma self-management, can be assertive about their rights, have good support systems as well as teachers and schoolmates who are trained and have positive perception of asthma. Tools such as inhalers and asthma diary were mentioned as necessary tools sufficient to help a child with asthma who is enrolled in a boarding school.

Children with uncontrolled asthma usually have poorer quality of life [16]. Uncontrolled asthma can lead to more frequent emergency department visits, hospitalizations, work absenteeism, school absenteeism, causing significant costs to families and society [17]. In a boarding school, uncontrolled asthma could increase costs to the school management. When asthma is well-controlled, the child will have normal lung function, minimal need for reliever medications as there

will be neither daytime symptoms nor night waking due to asthma, no limitation of activity, no airway obstruction, and no exacerbations which might prompt emergency care visits [18]. The Asthma Control Test^{TM} (ACT^{TM}) is a short, simple tool for identifying patients with poorly-controlled asthma [19, 20]. The test can be taken online or filled in hard copies, made available in the school clinic [21].

Commented [u9]: In what way(s) do these statements corroborate your own findings?

Commented [u10]: What is the relevance of this to your own findings?

To achieve asthma control, patients with asthma, and their caregivers, need to identify/avoid/eliminate their asthma triggers, adhere to the asthma action plan, adhere to prescribed/recommended therapy by health professionals during asthma reviews, use their inhalers appropriately, develop skills for asthma self-management, including use of ACT^{TM}, monitoring lung function with peak flow meters, and use of an asthma diary to track asthma management. Teachers in a boarding school with children with asthma, and the school management, can help enforce these by collaborating with the children, their schoolmates, parents/guardians, and health professionals. A study documented that parents should inform teachers about their children's written asthma action plan to guide the teacher during emergencies, rather than waiting for parents to pick up their children from school while taking no immediate action that can save a life [22].

Furthermore, a culturally tailored school-based intervention has been shown to reduce hospitalizations and emergency visits as well as reduce the days of restricted activity [23]. However, some participants believed that boarding schools are not appropriate for students with asthma, regardless of their severity and control. They cited concerns about the change of environment, exposure to allergens (including unknown ones), emotional unrest, lack of supervision, and the living conditions in most boarding schools. This opinion is similar to several studies that have reported that children with asthma are at increased risk of being bullied and victimized [24]. Another survey conducted in Nigerian primary and secondary schools leaves

much to be desired as majority of public schools in rural and poor urban slums have substandard environment that is suboptimal for not just students with asthma but non-asthmatics as well [25, 26].

Theme 2: Facilities necessary for boarding schools to cater for students with asthma

From the responses provided, it is evident that there is a consensus on the facilities required to manage students with asthma in boarding schools. This indicates that there are measures that must be put in place by boarding schools to cater for the needs of students with asthma. These facilities include: adequate and functional medical facilities with health professionals such as nurses, emergency asthma kits, and an ambulance to transport students to the hospital when necessary. Unfortunately, this seems not to be the case as assessment of school health services in Nigeria revealed poor and sub-standard health facilities in most public and even private schools. An assessment of asthma care services in Lagos State, Nigeria, revealed that only 16% out of 54 schools surveyed had a school clinic; only 7.4% had a school health worker; and none of the schools had facilities for asthma emergency care [10, 27].

Findings from this study also indicate that effective referral systems for severe emergencies are necessary, including retaining an agreement with a standard hospital or a pulmonologist. Additionally, the school must minimize exposure to environmental triggers like dust, pollen, and cold. It is also essential that the school and parents/guardians work together to provide proper communication, medication, and necessary resources to keep children with asthma safe in school. Training and re-training of staff and students to recognize asthma symptoms and provide help is crucial.

Theme 3: Outright rejection of children with asthma by ill-equipped boarding schools

Regarding the outright rejection of children with asthma by boarding schools, most respondents agreed that boarding schools should not simply reject students with asthma. Instead, boarding schools should provide the necessary facilities and resources to create an inclusive and supportive environment for all students, including those with asthma. Asthma is a common chronic condition, and with proper management and support, students with asthma can thrive academically and socially. Therefore, rejecting students with asthma is discriminatory and can limit their opportunities for personal and academic growth.

However, some respondents further noted that boarding schools should reject known asthmatics if they are not equipped to take care of them. This is to avoid any negative consequences that may arise from accepting students without adequate facilities to manage them. If a boarding school is not equipped to handle students with asthma, they should inform the parents or guardians and advise them to opt for day school. Some participants were of the opinion that even schools who do not have all it takes to manage such conditions in place could also accept such students, on the condition that they explain their shortcomings to the parents or guardians of these children. This could prevent their actions from being misconstrued as stigmatization of children with asthma and might ensure parents/guardians are held responsible for whatever happens to the child if they eventually decide to ~~enrol~~enroll.

The study under consideration was not devoid of limitations. It was conducted in a closed social media group, specific to members involved with promoting asthma awareness and care in communities. It is possible that this might have influenced the responses. Further studies can consider focus group discussions with participants being teachers, school students,

parents/guardians and school management.

These perspectives will generate well-rounded evidence and the attendant recommendations. As the prevalence of childhood asthma increases in Nigeria, considerations about its effective management, within the context of school systems, become increasingly topical. However, given that majority of the participants involved in this study were health professionals involved in asthma care, the opinions elicited in this study represent those of stakeholders who are knowledgeable about the disease state and the resources necessary for adequate management of asthma.

Conclusions

This study, in an attempt to address the issue of the appropriateness of boarding school for children with asthma, has identified children's age, autonomy, asthma management status, and the school's readiness as important considerations for the safe attendance of children with asthma at boarding schools. With childhood asthma being common, it is expedient that schools put measures in place to ensure that students with asthma can lead active lives. They should be able to learn in a safe environment without fear of stigmatization or lack of facilities/medications/expertise/referral systems, in emergencies. The concept of school health must be promoted according to public health guidelines. Health club should be encouraged in boarding schools.

Acknowledgements

The authors appreciate all members of the Asthma Awareness and Care Group (AACG) for their participation and responses which were the basis for the study. AACG also appreciates the Respiratory Pharmacists of Nigeria (RPN) which is a specialty group under the Clinical Pharmacists Association of Nigeria (CPAN). The case study used in this research was coined by Pharmacist Billy Shoaga of RPN/CPAN.

Was the study funded? Acknowledge the funders.

References

1. Serebrisky D, Wiznia A. Pediatric asthma: a global epidemic. Annals of Global Health. 2019;85(1):6. doi: 10.5334/aogh.2416.

2. Ferrante G, La Grutta S. The burden of pediatric asthma. Frontiers in Pediatrics. 2018;6:186. doi: 10.3389/fped.2018.00186.

3. Subbarao P, Mandhane PJ, Sears MR. Asthma: epidemiology, etiology and risk factors. Canadian Medical Association Journal. 2009;81(9):E181–E190. doi: 10.1503/cmaj.080612.

4. Kozyrskyj AL, Ernst P, Becker AB. Increased risk of childhood asthma from antibiotic use in early life. Chest. 2007;131(6):1753–1759.

5. Adeloye D, Chan KY, Rudan I, Campbell H. An estimate of asthma prevalence in Africa: a systematic analysis. Croatian Medical Journal. 2013;54(6):519-531.

6. Asher I, Pearce N. Global burden of asthma among children. International Journal of Tuberculosis and Lung Disease. 2014;18(11):1269-1278. doi: 10.5588/ijtld.14.0170.

7. Kuti BP. Schooling and Nigerian children with asthma: a review of challenges, risks and prospects. Nigerian Journal of Clinical Practice. 2021;24(9):1268-1276. doi: 10.4103/njcp.njcp_156_20.

8. Global Initiative for Asthma (GINA). Global strategy for asthma management and prevention (2022 update). Available from: https://ginasthma.org (Accessed on 1 May, 2023).

9. Edelu BO, Eze JN, Ayuk AC, Oguonu T. Prevalence and pattern of asthma exacerbation in children seen at the University of Nigeria Teaching Hospital, Enugu. Nigerian Journal of Paediatrics. 2016;43(2):78-82. doi: 10.4314/njp.v43i2.3.

10. Oyinlade OA, Ogunkunle OO, Olanrewaju DM. An evaluation of school health services in Sagamu, Nigeria. Nigerian Journal of Clinical Practice. 2014;17(3):336-342.

11. Ola BA, Ani C. School children's stigmatising attitude towards peers with asthma in Nigeria. Neuropsychiatrie de l'Enfance et de l'Adolescence. 2012;60(5):S229. https://doi.org/10.1016/j.neurenf.2012.04.535.

12. Williams B, Hoskins G, Pow J, Neville R, Mukhopadhyay S, Coyle J. Low exercise among children with asthma: A culture of over protection? A qualitative study of experiences and beliefs. British Journal of General Practice. 2010; 60(577):319-326.

13. Hauptman M, Phipatanakul W. The school environment and asthma in childhood. Asthma Research and Practice. 2015;1:12. doi: 10.1186/s40733-015-0010-6.

14. Magzamen S, Patel B, Davis A, Edelstein J, Tager IB. Kickin' asthma: school-based asthma education in an urban community. Journal of School Health. 2008;78(12):655-65. doi: 10.1111/j.1746-1561.2008.00362x.

15. Asthma Awareness and Care Group (AACG). Available on: https://www.asthmacaregroup.org.ng (Accessed on 1 May, 2023).

16. Banjari M, Kano Y, Almadani S, Basakran A, Al-Hindi M, Alahmadi T. The relation between asthma control and quality of life in children. International Journal of Pediatrics. 2018;2018:6517329. doi: 10.1155/2018/6517329.

17. Centers for Disease Control and Prevention. Asthma: uncontrolled asthma among children, 2012 – 2014. Available from: https://www.cdc.gov/asthma/asthma_stats/uncontrolled-asthma-children.htm (Accessed on 1 May, 2023)

18. Braido F. Failure in asthma control: reasons and consequences. Scientifica. 2013;2013:549252. doi: 10.1155/2013/549252.

19. Nathan RA, Sorkness CA, Kosinski M, Schatz M, Li JT, Marcus P, et al. Development of the asthma control test: a survey for assessing asthma control. Journal of Allergy and Clinical Immunology. 2004;113(1):59-65. doi: 10.1016/j.jaci.2003.09.008.

20. Liu AH, Zeiger R, Sorkness C, Mahr T, Ostrom N, Burgess S, et al. Development and cross-sectional validation of the Childhood Asthma Control Test. Journal of Allergy and Clinical Immunology. 2007;119(4):817-825. doi: 10.1016/j.jaci.2006.12.662.

21. GSK. Take the asthma control test. Available from: https://www.asthma.com/understanding-asthma/severe-asthma/asthma-control-test/ (Accessed on 1 May, 2023).

22. Withers AL, Green R. Transition for adolescents and young adults with asthma. Frontiers in Pediatrics. 2019;7:301. https://doi.org/10.3389/fped.2019.00301.

23. Schwei RJ, Johnson T, Matthews AK, Jacobs EA. Perceptions of negative health-care experiences and self-reported health behaviour change in 3 racial and ethnic groups. Ethn Health 2017;22(2):156-168. https://doi.org/10.1080/13557858.2016.1244621.

24. Gibson-Young L, Martinasek MP, Cluster M, Forrest J. Are students with asthma at increased risk of being a victim of bullying in school or cyberspace? Findings from the 2011 Florida youth risk behaviour survey. Journal of School Health. 2014:84(7):429-434. doi: 10.1111/josh.12167.

25. Olatunya O, Oseni S, Olaleye A.O, Olatunya A, Akani N. School health services in Nigeria: a sleeping giant? African Journal of Health Sciences. 2015;28(1):127-141.

26. Toma B, Oyebode T, Toma G.I.O, Agaba E. School health services in primary schools in Jos Nigeria. Open Science Journal of Clinical Medicine. 2014;2(3):83-88.

27. Adeyeye OO, Kuyinu YA, Ozoh OB. Assessment of the knowledge of teachers about asthma and the availability of facilities for asthma care in public secondary schools in Lagos Nigeria.

African Journal of Thoracic and Critical Care Medicine 2018;24(2):76-81. 10: 7196/AJTCCM.2018.v24i2.192.
