# Supplementary material for: Are boarding secondary schools suitable for students with asthma? An asynchronous online focus group discussion among members of an asthma awareness group
Source: PLoS One. 2024 Sep 6;19(9):e0304123. doi: 10.1371/journal.pone.0304123 (PMC11379369; doi:10.1371/journal.pone.0304123)
Supplement: Attachment — Submitted filename: (Response to Reviewers) - PONE-D-23-13651.docx [file pone.0304123.s002.docx]

**RESPONSES TO THE REVIEWERS**

**PONE-D-23-13651: Are boarding secondary schools suitable for students with asthma? A focus group discussion**

**RESPONSES TO REVIEWER 1**

**MAJOR COMMENTS**

**1. Sample Size and Participant Selection: The manuscript does not provide a clear rationale for the selection of the 19 respondents from the initial 150 participants. It is important to provide a justification for the final sample size and explain any potential biases or limitations associated with the selection process.**

***See page 8: Sample size and selection***

*Sample size was not calculated. All registered members of the AACG WhatsApp^®^ platform were eligible for participation. Convenience sampling technique, within a specified timeline, was employed. Comments were received from 19 participants who provided responses within the allotted timeframe for data collection.*

***See page 33: Limitations of the study***

*The study was conducted within the confines of a closed social media group, the Asthma Awareness and Care Group (AACG), which is predominantly made up of healthcare professionals and enthusiasts involved in asthma care. These participants may possess a higher level of knowledge and expertise regarding asthma management than the general population and may not fully represent a general perspective on the subject matter. Furthermore, the expertise and familiarity with the subject matter might have influenced their responses and recommendations. In addition, the study employed convenience sampling without calculating sample size. As a result, the sample size may not be sufficiently large or diverse to capture the wide range of opinions and experiences related to boarding schools and asthma management in Nigeria. Future studies should consider a more diverse range of participants.*

***Authors’ comments:*** *Sampling of participants for the study and the limitations associated with the selection process are included in the manuscript.*

**2. Thematic Analysis: While the manuscript mentions that thematic analysis was conducted, it lacks details about the specific steps and analytical framework used. Providing more information on the process of thematic analysis and the specific themes that emerged would enhance the transparency and rigor of the study. Additionally, it would be helpful to include a brief discussion on the validity of the findings.**

***See Page 8: Methods; Data analysis section***

*For the identification of a thematic framework, nine researchers independently acquainted themselves with the transcripts, acquiring a general feel for the data and noting emergent ideas on the margins. The researchers identified key themes in the transcript. This was similar to a study conducted on challenges and expectations of the Mental Capacity Act of 2005 where an in-depth qualitative methodology was adopted. Three a priori questions formed the thematic framework, based on the codes and sub-themes that were subsequently identified [19]. The thematic framework included three central themes: 1) appropriateness of boarding school for children with asthma; 2) facilities necessary for boarding schools to cater for children with asthma; 3) outright rejection of children with asthma by boarding schools.*

*For the “indexing” stage, the nine researchers were assigned different parts of the transcripts, which they iteratively read to identify ideas and assign codes to portions of text. This generated a coding framework. This process was repeated until no new codes were generated. Discrepancies between the nine researchers' codes were resolved through discussion and a unified coding framework was obtained [20]. These codes were initially identified under the three broad themes in the thematic framework, after which similar codes were clustered to form sub-themes.*

*In the “charting” step, these themes, subthemes and codes were presented in a chart and discussed at team meetings, enabling the re-ordering of the subthemes as necessary. This made it possible to extract relevant details from the data, including elements that may have been missed if a purely a priori approach was employed [21].*

*In the “mapping and interpretation” stages, the themes and subthemes were tabulated alongside exemplar quotes from participants. This process produced a 'tree structure' featuring interconnected themes, subthemes, and supporting quotes. Finally, these identified ideas were interpreted to answer the research questions and identify areas for public health action.*

***Authors’ comments:*** *Modifications have been made to this section, in line with the Reviewer’s comments.*

**3. Generalizability: The manuscript should acknowledge the limitations regarding generalizability. The study was conducted within a specific social media group and included participants who are actively involved in asthma care. Therefore, the perspectives presented may not fully represent the broader population. It is recommended to discuss the limitations and suggest future research directions to include a more diverse range of participants.**

***See page 33: Limitations of the study***

*The study was conducted within the confines of a closed social media group, the Asthma Awareness and Care Group (AACG), which is predominantly made up of healthcare professionals and enthusiasts involved in asthma care. These participants may possess a higher level of knowledge and expertise regarding asthma management than the general population and may not fully represent a general perspective on the subject matter. Furthermore, the expertise and familiarity with the subject matter might have influenced their responses and recommendations. In addition, the study employed convenience sampling without calculating sample size. As a result, the sample size may not be sufficiently large or diverse to capture the wide range of opinions and experiences related to boarding schools and asthma management in Nigeria. Future studies should consider a more diverse range of participants.*

***Authors’ comments:*** *Modifications have been made to highlight the limitations regarding generalizability of the findings of the study. A statement, on the need for future studies to include a more diverse range of participants, has been added.*

**4. Recommendations and Implications: The manuscript briefly mentions recommendations for boarding schools to create an inclusive and supportive environment for students with asthma. However, these recommendations should be further developed and expanded upon to provide actionable suggestions for policymakers, school administrators, and healthcare professionals. Consider discussing specific strategies for improving asthma management in boarding schools, such as the training of staff, collaboration with healthcare providers, and development of asthma-friendly environments.**

***See Page 32, 33***

*This study is an attempt to evoke thought process on whether children with asthma should be in boarding schools. It suggests that the suitability of boarding schools for children with asthma is dependent on many factors. Boarding schools should create an inclusive and supportive environment for students with asthma.*

*Boarding school administrators should ensure that they have adequate facilities to manage patients with asthma. This includes a functional sick bay and health professionals who would be available whenever needed. The sick bay should have medicines for the management of acute and chronic asthma, as well as asthma devices such as peak flow meters and nebulisers.*

*The boarding school should have links with registered hospitals that can handle referrals. There should be collaborative efforts amongst the teachers, students, parents and school administrators to ensure students with asthma have good quality of life while at school.*

*Asthma education programmes should be conducted for all active players (students, parents, teachers, school administrators). There should be trainings and re-trainings for staff and active players who can serve as first-responders if there is an asthma attack.*

*The boarding school should have asthma-friendly environments. All possible triggers for asthma should be eliminated.*

*Policy makers and those who approve schools to take boarding students should ensure that the facilities and environments are suitable for patients with asthma. Lists of items that need to be put in place should be provided to school administrators. Policy makers should form a think tank using this study as baseline with a view to providing policy framework that will guide the educational sector to make important changes to the boarding schools that would host students with asthma.*

*Future studies should consider quantitative assessments. Questionnaires could be administered to a larger sample size to quantify the prevalence of specific opinions and perceptions related to boarding schools for children with asthma. Longitudinal studies could also be conducted to provide valuable insights into the dynamics of opinions regarding boarding schools for children with asthma and allow for a deeper understanding of the factors influencing these perspectives. Mixed-method analysis could be done to better understand the experiences and health outcomes of children with asthma attending boarding schools in comparison to those attending day schools.*

*Building upon the findings of this study, future research could focus on developing and implementing interventions aimed at improving the management and support systems for children with asthma who are in boarding schools. Evaluating the effectiveness of interventions, such as asthma education programmes for teachers and students, improved medical facilities and resources, and strategies to reduce stigma and promote inclusivity could provide evidence-based recommendations for creating asthma-friendly environments in boarding schools.*

***Author’s comments:*** *Modifications have been made, in line with the suggestions of the Reviewer.*

**MINOR COMMENTS**

**1. Geographical Context: Consider providing additional contextual information on the Nigerian education system, including the prevalence of boarding schools and their characteristics. This would help readers better understand the specific context in which the study was conducted.**

***See Page 5: Introduction section***

*Nigeria currently follows the Universal Basic Education (9-3-4 system of education), a transition from the 6-3-3-4 system [7]. The 9-3-4 system entails nine years of basic education, three years of senior secondary education, and four years of tertiary education. The curriculum for the 9-3-4 system of education was designed to meet the Millenium Development Goals (MDGs) by 2020 [7]. Students can be in boarding schools from the seventh to ninth year of basic education and during the three years of senior secondary education. There is an option of having children going from home (day students) or living and studying in the school during the school term (boarders) [8].*

*In Nigeria, 5 of every 100 adolescents in Nigeria who are school children, especially those in secondary schools, are in boarding schools [9]. Many parents, lean towards the option of boarding schools as it helps children build independence, limits access to distracting technology, and provides an established learning community where they can easily form study groups [10].*

***Author’s comments:*** *Modifications have been made, in line with the Reviewer’s comments.*

**2. Limitations and Future Research: While this study provides valuable insights into the perceptions and opinions regarding boarding schools for children with asthma, it is important to acknowledge its limitations and identify areas for future research.
(a) Quantitative Assessment: This study adopted a qualitative design using focus group discussions. While qualitative methods are valuable in exploring in-depth perspectives and experiences, future research could complement these findings with quantitative assessments. Surveys or questionnaires could be administered to a larger sample size to quantify the prevalence of specific opinions and perceptions related to boarding schools for children with asthma. This would allow for statistical analysis and a more comprehensive understanding of the overall attitudes towards this topic.
(b) Longitudinal Studies: The current study provides a snapshot of participants' opinions at a specific point in time. Conducting longitudinal studies would enable the examination of changes in attitudes and perceptions over time. This would provide valuable insights into the dynamics of opinions regarding boarding schools for children with asthma and allow for a deeper understanding of the factors influencing these perspectives.
(c) Comparative Analysis: To gain a better understanding of the suitability of boarding schools for children with asthma, future research could compare the experiences and outcomes of children with asthma attending boarding schools versus day schools. This comparative analysis would shed light on the potential advantages and disadvantages of each educational setting and help inform policymakers, school administrators, and parents/guardians in making well-informed decisions.
(d) Intervention Studies: Building upon the findings of this study, future research could focus on developing and implementing interventions aimed at improving the management and support systems for children with asthma in boarding schools. Evaluating the effectiveness of interventions, such as asthma education programs for teachers and students, improved medical facilities and resources, and strategies to reduce stigma and promote inclusivity, would provide evidence-based recommendations for creating asthma-friendly environments in boarding schools.**

**See Page 33:**

*Future studies should consider quantitative assessments. Questionnaires could be administered to a larger sample size to quantify the prevalence of specific opinions and perceptions related to boarding schools for children with asthma. Longitudinal studies could also be conducted to provide valuable insights into the dynamics of opinions regarding boarding schools for children with asthma and allow for a deeper understanding of the factors influencing these perspectives. Mixed-method analysis could be done to better understand the experiences and health outcomes of children with asthma attending boarding schools in comparison to those attending day schools.*

*Building upon the findings of this study, future research could focus on developing and implementing interventions aimed at improving the management and support systems for children with asthma who are in boarding schools. Evaluating the effectiveness of interventions, such as asthma education programmes for teachers and students, improved medical facilities and resources, and strategies to reduce stigma and promote inclusivity could provide evidence-based recommendations for creating asthma-friendly environments in boarding schools.*

***Author’s comments:*** *Modifications have been made, in line with the Reviewer’s comments.*

**RESPONSES TO REVIEWER 2**

1. The manuscript is very rich in vital scientific information that is of immense benefit to public and school health, which has been a neglected tropical issue. However, my major concern is the fact that out of 150 participants only 19 respondents' responses were available and used for discussions and drawing conclusions. The question now is, are these 19 responses sufficient to make the conclusion seen in this manuscript?

**See Page 33 (Conclusion):**

*This study, in an attempt to address the issue of the appropriateness of boarding school for children with asthma**, has identified children’s age, autonomy, asthma management status, and the school’s readiness, as important considerations for the safe attendance of children with asthma at boarding schools.*

***Author’s comments:*** *The small sample size is included as one of the limitations of the study. The study provides a baseline for future studies. It is an attempt to generate some insights and thought-provoking discussions on the subject matter.*

**2. Secondly, why was sample size not determined for the study? This would have ensured that more responses and statistically significant population responses for concrete conclusion is gotten.**

**See Page 8 (Sample size and selection):**

*Sample size was not calculated. All registered members of the AACG WhatsApp^®^ platform were eligible for participation. Convenience sampling technique, within a specified timeline, was employed. Comments were received from 19 participants who provided responses within the allotted timeframe for data collection.*

***Authors’ comments:*** *All registered members of the group were eligible for participation. The total population was wieldy. Due to the manageable total population and the possibility of non-participation by some members, it was made open to all but within a specified timeline.*

**3. Thirdly, you stated that majority of respondents were is support of allowing children with asthma go to boarding school. What's your definition of majority? It is better you state the majority in numerical terms.**

**See Page 29:**

*Majority of the participants (61.5%) consented that children with asthma can be allowed to attend boarding schools with strict conditions.*

***Authors’ comments:*** *The percentage has been included.*

**4. The use of social media and WhatsApp differently is a mere repetition.
The method(s) used were not properly cited and where modifications exist were not spelt out.**

**See Page 8 (Methods; Data analysis section)**

*For the identification of a thematic framework, nine researchers independently acquainted themselves with the transcripts, acquiring a general feel for the data and noting emergent ideas on the margins. The researchers identified key themes in the transcript. This was similar to a study conducted on challenges and expectations of the Mental Capacity Act of 2005 where an in-depth qualitative methodology was adopted. Three a priori questions formed the thematic framework, based on the codes and sub-themes that were subsequently identified [19]. The thematic framework included three central themes: 1) appropriateness of boarding school for children with asthma; 2) facilities necessary for boarding schools to cater for children with asthma; 3) outright rejection of children with asthma by boarding schools.*

*For the “indexing” stage, the nine researchers were assigned different parts of the transcripts, which they iteratively read to identify ideas and assign codes to portions of text. This generated a coding framework. This process was repeated until no new codes were generated. Discrepancies between the nine researchers' codes were resolved through discussion and a unified coding framework was obtained [20]. These codes were initially identified under the three broad themes in the thematic framework, after which similar codes were clustered to form sub-themes.*

*In the “charting” step, these themes, subthemes and codes were presented in a chart and discussed at team meetings, enabling the re-ordering of the subthemes as necessary. This made it possible to extract relevant details from the data, including elements that may have been missed if a purely a priori approach was employed [21].*

*In the “mapping and interpretation” stages, the themes and subthemes were tabulated alongside exemplar quotes from participants. This process produced a 'tree structure' featuring interconnected themes, subthemes, and supporting quotes. Finally, these identified ideas were interpreted to answer the research questions and identify areas for public health action.*

***Authors’ comments:*** *The Reviewer’s comments have been addressed. Modifications have been made to highlight the step-by-step processes used in the study.*

**5. Many statements made in the discussion were not related to corroborate the work of others. It was a mere hanging statement**

**See page 29, 30:**

*For instance, the asthma must be well-controlled based on the Asthma Control Test^TM^ (ACT^TM^), the children must be well-equipped by their parents on skills for asthma self-management such as proper inhaler and asthma diary use, they must be assertive about their rights, and have good support systems in form of teachers and schoolmates who are trained and have positive perception of asthma. A study opined that parents should inform teachers about their children’s written asthma action plan to guide teachers during emergencies, rather than waiting for parents to pick up their children from school while taking no life-saving actions [22]. Another study documented that school-based educational interventions led to improvement in teachers’ knowledge, self-management and health outcomes of asthma students [23]. All these are to abate the poorer quality of life, frequent emergency department visits, hospitalizations, and school absenteeism that emanates from uncontrolled asthma [24, 25].*

*Some participants believed that boarding schools are not appropriate for students with asthma, regardless of the asthma severity and control. They cited concerns about the change of environment, exposure to allergens (including unknown ones), emotional unrest, lack of supervision, and the living conditions in most boarding schools in Nigeria.* *This opinion is similar to several studies that have reported that children with asthma are at increased risk of being bullied and victimized in school [26]. Another survey conducted in Nigerian primary and secondary schools leaves much to be desired as majority of public schools in rural and poor urban slums have substandard environment that is suboptimal for not just students with asthma but non-asthmatics as well [27, 28].*

**Authors’ comments:** Modifications have been made in the Discussion section, in line with the Reviewer’s comments.

**6. Some of the statements were also just made without linkages**

**See Page 29:**

*Majority of the participants (61.5%) consented that children with asthma can be allowed to attend boarding schools with strict conditions. For instance, the asthma must be well-controlled based on the Asthma Control Test^TM^ (ACT^TM^), the children must be well-equipped by their parents on skills for asthma self-management such as proper inhaler and asthma diary use, they must be assertive about their rights and have good support systems in form of teachers and schoolmates who are trained and have positive perception of asthma. A study opined that parents should inform teachers about their children’s written asthma action plan to guide the teacher during emergencies, rather than waiting for parents to pick up their children from school while taking no immediate action that can save a life [22] and another study documented that school-based educational intervention led to improvement in teachers’ knowledge, self-management and health outcomes of asthma students [23]. All these are to abate the poorer quality of life, frequent emergency department visits, hospitalizations, and school absenteeism that emanates from uncontrolled asthma [24, 25].*

**Authors’ comments:** *Modifications have been made in the Discussion section, in line with the Reviewer’s comments.*

**7. If the research was funded, the sponsors need to be acknowledged**

**See Page 34: Funding Statement**

*This research received no funding.*

***Authors’ comments:*** *The research received no funding. This statement has been included in the manuscript.*
