# Supplementary material for: Are boarding secondary schools suitable for students with asthma? An asynchronous online focus group discussion among members of an asthma awareness group
Source: PLoS One. 2024 Sep 6;19(9):e0304123. doi: 10.1371/journal.pone.0304123 (PMC11379369; doi:10.1371/journal.pone.0304123)
Supplement: Attachment — Submitted filename: (RESPO~1.DOC [file pone.0304123.s003.DOC]

**RESPONSES TO THE REVIEWERS**

**PONE-D-23-13651R1: Are boarding secondary schools suitable for students with asthma? A focus group discussion**

**COMMENTS AND RESPONSES**

**1. In the study title, possibly add the type of FGD or participants.**

***See page 1, Lines 1 - 3: Study title***

*Are boarding secondary schools suitable for students with asthma? An asynchronous online focus group discussion among members of an asthma awareness group*

***Authors’ comments:*** *The type of focus Group Discussion and participants have been included in the title.*

**2. Indicate “parents or caregivers”, for the rest of the document, the authors should indicate that reference to parents includes caregivers.**

***See page 3, Lines 43, 44***

***See page 4, Line 90***

***See page 6, Line 97***

***See page 29, Theme 1, Second Paragraph***

***Authors’ comments:*** *Parents or caregivers have been indicated all through the document.*

**3. The authors note that demographic data were collected – any more apart from age and perhaps profession? It could have been useful in analysis.**

***Authors’ comments:*** *The only demographic data collected were age, gender, and profession. The analysis was focused on generating themes from the different responses. The authors did not deem it necessary to request for more demographic data as a test of association between demographic data and themes was not included as a study objective.*

*If it is highly-important, it can be included as a limitation of the study.*

*We have included the Participant Number (e.g., P1, P2 etc.) in the Tables.*

**4. I suggest that participants refer to the 19 not `150; 150 were contacted and 19 responded. L48 – there is no evidence that all the 150 members participated.**

***See page 3, Line 49: Abstract***

*The group comprised 150 registered members.*

***See page 3, Line 52: Abstract***

***Results:*** *Out of the 150 eligible members, there were responses from only 19 participants.*

***Authors’ comments:*** *Corrections have been made in the Abstract section and Main Text, as suggested, to eliminate ambiguities. Although the 150 registered members in the WhatsApp group received the messages (there is evidence that it was delivered to al)l, only 19 members responded with comments.*

**5. L150-155 – This looks more like a vignette.**

***See page 3, Line 50: Abstract***

*… based on a vignette comprising three questions.*

***Page 7, Line 121: Study design***

*The vignette was posted in the Group, without previous publicity.*

***See page 8, Line 151: Data collection***

*This vignette was posted on the AACG WhatsApp^®^ platform:*

***Authors’ comments:*** *Corrections have been made in the Abstract section and Main Text.*

**6. I wonder whether it is appropriate to quantify such small numbers (61.5%) when it is a purely qualitative study.**

***See Page 3, Line 62***

***See Page 29, Theme 1, Second Paragraph***

***Authors’ comments:*** *The percentage was not previously stated. It was included based on the request by one of the Reviewers. It provides an idea about the proportion of respondents who supported that statement. Maybe the inclusion or exclusion of the percentage figure should be left for the discretion of the Editor.*

**General Response**

*In addition, typographical and grammatical errors have been corrected in this revision. All edits are in red fonts.*
